# Expert insights: 10 key questions on managing common infections in cancer care in India

**DOI:** 10.3332/ecancer.2025.1971

**Published:** 2025-08-19

**Authors:** Lingaraj Nayak, Gaurav Salunke, Trupti Gilada, Sukhada Savarkar, Bindiya Salunke, Sanjay Biswas, Vanita Noronha, Atul Kulkarni, Manju Sengar, Akshay Baheti, Pradnya Samant, Anant Gokarn, Anuradha Mehta, Chetan Dhamne, Keerthna Batyala

**Affiliations:** 1 Department of Medical Oncology, Tata Memorial Hospital, Mumbai, India; 2 Homi Bhabha National Institute(HBNI), Mumbai, India; 3 Department of Microbiology, Tata Memorial Hospital, Mumbai, India; 4 Department of Infectious Diseases, Unison Medicare and Research Centre, Mumbai, India; 5 Department of Anaesthesiology and Critical Care, Tata Memorial Hospital, Mumbai, India; 6 Department of Radiodiagnosis, Tata Memorial Hospital, Mumbai, Mumbai, India; 7 Bone Marrow Transplant Unit, ACTREC, Tata Memorial Hospital, Mumbai, India; 8 Department of Paediatric Oncology, Tata Memorial Hospital, Mumbai, India

**Keywords:** cancer, infections, MDR, immunosuppression, sepsis

## Abstract

Cancer patients are at a heightened risk of infections due to immunosuppression from chemotherapy, radiotherapy and the malignancy itself, contributing to increased morbidity and mortality. Effective infection management in this vulnerable population requires a systematic and timely approach to diagnosis and treatment. This review addresses ten critical questions concerning the management of infections in cancer patients, synthesising insights from clinical guidelines, expert opinions and current evidence.

The review begins by discussing the optimal diagnostic workup for neutropenic patients, including investigations, risk stratification and treatment approaches for various neutropenia-specific syndromes. It further explores the principles of antibiotic escalation and de-escalation for gram-negative infections, emphasising the need for tailored therapeutic strategies. Advances in microbiological diagnostics, such as early detection methods and understanding resistance mechanisms in gram-negative organisms and *Clostridioides difficile* infections, are analysed in dedicated sections. The role of radiological investigations, which remain the cornerstone for diagnosing infections in immunocompromised patients, has been addressed. Catheter-related blood stream infection and the role of surveillance culture are explored in the final section. By addressing these critical questions, this review provides oncology clinicians with practical, evidence-based guidance for preventing, diagnosing and managing infections in cancer patients. The insights presented aim to enhance clinical outcomes and ensure patient safety in this high-risk population.

## Background

Patients with cancer are at an increased risk of developing infections due to a combination of factors, including immunosuppression from chemotherapy, radiation therapy and the underlying malignancy itself. Infections in this population can lead to significant morbidity and mortality, often complicating the course of cancer treatment. Understanding the types of infections based on newer methods of diagnosis is essential for timely diagnosis and management. This paper aims to address ten key questions related to the management of infections in patients with active malignancy, providing an overview based on current evidence and expert insights.

## Method

### Study design

This is a question-and-answer-based review paper synthesising information from clinical guidelines and expert opinions on infections in cancer patients.

### Selection of questions

The development of this manuscript involved a systematic process to identify and curate 10 key questions addressing the management of common infections in cancer care. The following steps were undertaken to prepare the manuscript.

### Identification of clinical gaps

Based on the literature review, gaps in clinical knowledge and areas requiring practical guidance were identified. Emphasis was placed on questions that reflect real-world dilemmas faced by oncology clinicians, such as infection prevention, diagnosis and treatment in immunocompromised patients.

### Consultation with experts

An expert panel of oncologists, infectious disease specialists, microbiologist, intensivists and healthcare professionals actively involved in cancer care was convened. Each author was asked to provide a list of commonly encountered and challenging questions related to infections in their clinical practice. Based on priority and clinical significance, 10 important questions were selected for this consensus document.

## Question No- I:- What is the optimal work-up in patients with neutropenia and suspected infection and how do you risk stratify?

## Answer

## History taking

I.1. a Include the type of chemotherapy, prior infections and their resistance patterns and comorbidities.

## Clinical examination

I.2. a Focus on areas commonly affected in neutropenic patients: oral mucosa, perianal area, skin, catheter insertion sites, GI and lung focus.

I.2. b Identification of neutropenic-specific syndrome(Table 1) is important in tailoring the investigation and management early.

## Investigations

I.3. a A minimum of one set of blood cultures should be sent, including samples from a peripheral line and existing indwelling intravenous lines.

I.3. b If a focus of infection is suspected, bacteriological cultures from the specific site will aid in tailoring antibiotic decisions after 24–48 hours.

I.3. c The roles of CRP and procalcitonin in neutropenic patients remain areas of ongoing research. Routine use of these biomarkers for prognostic and predictive purposes is not recommended.

I.3. d A non-contrast CT scan of the chest is strongly recommended as a baseline investigation in neutropenic patients with clinical findings or non-response to first-line antibiotics after 48 hours.

## Risk stratification

Assessment of the risk for complications of severe infection should be undertaken at the presentation of fever. Risk stratification helps determine the type of empirical antibiotic therapy (oral versus intravenous), treatment venue (inpatient versus outpatient) and duration of antibiotic therapy. Key considerations include the anticipated duration and degree of neutropenia, the chemotherapy regimen used, type of cancer (hematolymphoid or solid tumour), disease remission status and significant medical comorbidities.

## I.4. a High-risk patients

Patients with any of the following factors require initial hospitalisation for empirical antibiotic therapy:

**• Host factors**

- Age > 60 years
- Comorbidities such as uncontrolled diabetes, cardiac or renal conditions or obstructive lung disease
- History of ICU admission or multidrug-resistant (MDR) sepsis
- In paediatrics, down syndrome and other immune deficiency.

**• Disease and Therapy**

e. Acute leukemia patients on induction chemotherapy
f. Expected prolonged neutropenia (>7 days)
g. High-risk regimens (e.g., OGS-2012, EFT-2001, DCF regimen, high-dose cytarabine consolidation, Cladribine for hairy cell leukemia, BEP, TIP, VbIP, EMACO/EP regimen, COG protocol for Ewing's Sarcoma, MAP Protocol for OGS, VIP Protocol for GCT)
h. Profound neutropenia (absolute neutrophil count [ANC] < 100 cells/mm³)

**• Comorbidity/Focus of infection**

i. Hypotension (BP < 100/60 mmHg or poor pulse volume)
j. Hypoxia (SpO2 < 94%)
k. Specific foci of infection (e.g., *pneumonia*, catheter-related infections, chest tubes, nephrostomy tubes/DJ stents)
l. Features suggestive of enterocolitis
m. Depressed level of consciousness
n. Evidence of hepatic or renal insufficiency

## I.4. b Low-risk patients

Patients without any high-risk features after careful selection may be candidates for outpatient empirical antibiotic therapy with daily follow-up.

## Question No – II :- What is escalation and de-escalation approach for gram-negative infection?

Answer:-

## II.1. Definitions

- **Antibiotic escalation**: Refers to the addition of another antibiotic or switching to a broader-spectrum antibiotic whenever there is clinical deterioration or laboratory evidence suggesting inadequacy of the initial coverage [3].
- **Antibiotic de-escalation**: Refers to deliberate narrowing or stopping of antibiotic therapy based on clinical improvement and laboratory evidence, if a non-infectious syndrome is suspected [2] including identification of the causative organism and its sensitivity.

## II.2. Steps for good practice in antibiotic use

### II.2.a. Initial empirical therapy

- Suspected infection/sepsis should prompt initiation of empirical broad-spectrum antibiotics based on the site of infection (e.g., blood, respiratory, intra-abdominal, soft tissue or urinary tract) and the origin of the sample (Outpatient Department (OPD) versus inpatient department (IPD)) and hospital antibiogram [1, 2].
- Table 2 gives the overlook about the emperical antibiotic therapy based on site of infection and origin of sample.

### II.2.b. Sample collection

- Collect appropriate samples for culture and sensitivity before initiating antibiotics.

## II.3. Rapid diagnostic tests

- Utilise rapid tests like gram stains to guide the initial choice of empirical therapy.

## II.4 Regular reassessment:

- Reassess antibiotic therapy regularly to ensure appropriateness of dose, route, indication and patient improvement.

### II.4 a Review after 48–72 hours

- Review clinical and microbiological data to guide further antibiotic management.

### II.4. b Continuing empirical therapy

- Continue empirical treatment if the pathogen is sensitive to the drug, culture is negative and the patient responds clinically.

### II.4. c Antibiotic escalation

- Consider escalation if:
  - Persistent or worsening infection leading to hypoxia, hypotension or organ dysfunction despite empirical therapy.
  - New evidence suggests inadequately covered pathogens (clinical, microbiological or imaging evidence).
  - Persistence of fever is not an indication for antibiotic escalation if patient is clinically stable.

### II.4. d Antibiotic de-escalation:

- Consider de-escalation if:
  iv. Patient shows clinical stability or improvement in infection markers.
  v. Microbiological evidence identifies a pathogen susceptible to narrower-spectrum agents.
  vi. A non-infectious cause of the clinical condition is diagnosed.
  vii. Redundant gram-negative or anaerobic coverage is present.
  viii. Patient stabilises clinically and can be switched from intravenous to oral antibiotics.
- **Avoid prolonged therapy**: The duration of antibiotics should be driven by the site of infection, blood culture and clinical status.

### II.4. e. Antibiotic selection

- **Community-acquired infections (CAIs)**
  - For patients from the community (not hospitalised) with minimal risk of multidrug-resistant pathogens, broad-spectrum antibiotics are typically unnecessary.
- **Hospital-acquired infections (HAIs)**
  - For hospitalised patients, there is a higher probability of healthcare-associated or nosocomial infections caused by resistant or multidrug-resistant pathogens such as extended-spectrum beta-lactamase (ESBL)-producing Enterobacteriaceae, MRSA, Pseudomonas and Acinetobacter. Consider broader-spectrum antibiotics such as:
    - Carbapenems
    - Beta-lactam/beta-lactamase inhibitors (BL + BLI)
    - Vancomycin/Teicoplanin
    - Polymyxins
    - Ceftazidime-Avibactam (CZA) with Aztreonam

### II.4. f. Empirical treatment recommendations

- For CAIs
  - Initiate empirical treatment based on common pathogens. If no improvement is seen within 48 hours, escalate to antibiotics typically used for HAIs until microbiological evidence is available (Table 2).
- For HAIs
  - If no improvement occurs with initial empirical treatment, consider the possibility of more resistant organisms such as *Pseudomonas* spp., *Acinetobacter* spp. or Vancomycin resistant Staphylococcus aureus/Vancomycin resistant Enterococci (VRSA/VRE) and adjust treatment accordingly.

## Question No – III :- Methods of susceptibility testing for gram-negative infections and their limitation.

Answer :- For determining the most appropriate course of treatment for gram-negative infections, antimicrobial susceptibility testing is crucial. Several methods are used, each with special advantages and disadvantages (Table 3).

## Question No – IV:- What are the indications and methods of doing ‘point of care’ resistant gene testing in gram-negative infections?

**Answer:-**Point-of-care (POC) resistance gene testing has shown considerable promise in the management of gram-negative infections, providing swift identification of antimicrobial resistance (AMR) genes. Research indicates its significance in enhancing therapeutic outcomes and refining antimicrobial stewardship. Table 4 depicts the various indication where point of care testing is indicated. Overview of POC tests for resistance gene detection has been briefed in Table 5.

Overview of POC tests for resistance gene detection has been briefed in Table 5.

Innovations in technology have made POC platforms like **Cepheid GeneXpert** and **BioFireFilmArray** reliable for rapid testing. According to Charnot-Katsikas *et al* [4], these systems have high sensitivity (90%–95%) and specificity (95%–99%), detecting important resistance genes in 1–2 hours as opposed to the 24–72 hours needed for culture-based techniques. This short turnaround time enables prompt tailored therapy and avoids unnecessary broad-spectrum antibiotic use by 25%–30% [5]. They are especially beneficial in ICUs, where infections with multidrug-resistant (MDR) organisms like *Klebsiella pneumoniae*, *Pseudomonas aeruginosa* and *Acinetobacter* are common. Moreover, rapid detection of ESBL and carbapenemase genes facilitates managing antimicrobial resistance more effectively.

While advantages exist, challenges like high costs limit its accessibility in resource-limited environments. Additionally, POC testing focuses only on the detection of resistant genotypes and does not provide any assessment of phenotypic patterns, thus requiring additional confirmation for comprehensive data.

In summary, evidence supports the use of POC resistance gene testing as a groundbreaking tool in managing gram-negative infections. Table 4** provides an overview of POC tests for resistance gene detection, highlighting their technology, features and clinical applications.** Its incorporation into routine clinical workflows will significantly enhance infection management in high-risk and resource-limited settings, facilitating rapid, precise and economically viable outcomes.

## Question No – V:- How will the decision of antibiotic choice be affected based on results of resistant gene testing?

**Answer:-** The information from antibiotic resistance gene testing is crucial for prompt initiation of the most effective targeted therapy and to avoid empiric use of broad-spectrum antibiotics. The choice of antibiotic is based on the site of infection, susceptibility data (if available) and host factors like organ dysfunction and so on. In resistant gram-negative infections, a combination therapy is recommended instead of monotherapy.

Case studies based on real world scenarios where point-of-care testing can help in the selection of appropriate antibiotics early leading to better outcome has been explained in appendix-A.

**Advances in treatment strategies for multidrug-resistant pathogens: focus on New Delhi metallo-β lactamase (NDM) and carbapenemase-producing organisms**

**1. Ceftazidime-Avibactam and Aztreonam for NDM-Producing Organisms-** The combination of **ceftazidime-avibactam** and **aztreonam** has shown efficacy against infections caused by **NDM-producing Enterobacterales**. This regimen capitalises on avibactam’s inhibition of serine β-lactamases, enabling aztreonam to remain effective against metallo-β-lactamases like **NDM**. Clinical studies have reported favorable outcomes with this combination, positioning it as a viable treatment option for such resistant infections [6].

**2. Treatment options for carbapenemase-producing organisms**

**Colistin** and **tigecycline** have traditionally been first-line agents for treating infections caused by carbapenemase-producing organisms. However, their efficacy remains uncertain, particularly when used in combination with other agents. More recently, several new agents have been approved for clinical use or are nearing late-stage clinical development. These include **ceftazidime-avibactam** +/- **aztreonam**, **ceftolozane-tazobactam**, **meropenem-vaborbactam**, **imipenem-cilastatin-relebactam**, **plazomicin**, **eravacycline** and **cefiderocol**. Additionally, **fosfomycin** has been redeveloped in a new intravenous formulation. Data on the clinical efficacy of these newer agents specific to infections caused by carbapenem-resistant pathogens are emerging, with early evidence favoring these new treatments over previously available options [7, 8].

**3. Colistin minimum inhibitory concentration (MIC) variability by contemporary methods**

Determining the **MIC** of **colistin** remains challenging due to variability among testing methods, particularly with automated systems, which may fail to reliably detect colistin resistance. **Broth microdilution**, the recommended reference method for colistin susceptibility testing, ensures accurate and consistent results, but it is labor-intensive. The diverse mechanisms and genes involved in colistin resistance hinder the rapid detection of resistance through molecular biology techniques. Combining newer phenotypic and genotypic testing methods is essential for a comprehensive understanding of colistin resistance, especially for strains carrying **mcr genes** [9].

## Question No –VI:-What is the best modality for Clostridioides difficile test? How do you compare between various methods?

Answer:- Peterson and Robicsek [10] conducted a prospective study in 2009 to examine the role of susceptibility testing in guiding clinical decision-making for *C. difficile* infections. Taking into consideration the balance between sensitivity, specificity and clinical outcomes, they recommended algorithms, combining both molecular and immunoassay methods to enhance diagnostic accuracy. Clinical guidelines [11] recommend an algorithm-based approach, by integrating molecular methods, such as NAATs, along with toxin detection assays for susceptibility testing. The objective was to improve isolation measures and infection control while minimising unnecessary antibiotic usage by separating colonisation from active infection.

The data collectively support the need for novel and integrated susceptibility testing methods to improve diagnosis accuracy and clinical management of *C. difficile* infections. Table 7 depicts Various methods for C difficile detection.

## Question No- VII :-Indications and utility of radiological investigations in immunocompromised patients for selection of antibiotics/antifungals or antiviral?

Answer:- The American College of Radiology Appropriateness Criteria, the Guideline for Management of Fever and Neutropenia in Children With Cancer and Hematopoietic Stem-Cell Transplantation Recipients by the Society of Clinical Oncology for imaging and the COG Diagnostic Imaging Committee/SPR Oncology Committee White Paper label the following studies as appropriate for initial evaluation of patients with febrile neutropenia [12–14].

- Chest radiograph (in patients with respiratory signs or symptoms – strong recommendation).
- CT chest without or with IV contrast, for patients with prolonged (>96 hours) febrile neutropenia when there is a concern for invasive fungal disease (strong recommendation).
- CT paranasal sinuses without or with IV contrast to not be performed without localising signs or symptoms (weak recommendation) as abnormal findings are commonly seen incidentally as well and cannot differentiate invasive from non-invasive fungal disease.
- FDG PET/CT (moderate recommendation)

Specific signs like the halo sign (central nodular consolidation surrounded by ground glass opacity) and the air crescent sign (intracavitary nodular soft tissue with a crescentic collection of air that separates it from the wall of the cavity) help in the diagnosis of angioinvasive fungal infection [15]. Ground glass opacities are more common in atypical bacterial or viral infections or *Pneumocystis jirovecii,* while lobar consolidation and effusions are more common with bacterial infection [16]. Mixed infections can, however, often complicate the picture in clinical practice and correlation with clinical and laboratory findings is always necessary.

## Question No- VIII :-What is catheter-related blood stream infection and how to treat CRBSI?

Answer:- CRBSI can be diagnosed if there is bloodstream infection (BSI) in a patient with an intravascular device who has more than one positive blood culture obtained from a peripheral vein, has clinical features suggestive of infection and no other apparent known source for the BSI.

### VIII.1. Recommendations for diagnosis

For definitive diagnosis of CRBSI, paired blood samples, drawn from the catheter hub and a peripheral vein, should be cultured before initiation of antimicrobial therapy. If a blood sample cannot be taken from a peripheral vein, it is recommended that ≥2 blood samples should be drawn through different catheter lumens. If the catheter is already removed for suspected CRBSI; then the catheter tip cultures should be performed [17].

One of the following should be present for diagnosis of CRBSI:

VIII 1.1. Isolation of the same organism from a quantitative blood culture drawn through the catheter hub and from a peripheral vein with a ratio of 3:1colony-forming units (cfu)/ml of blood (catheter versus peripheral blood).

VIII 1.2. Differential time to positivity: positive blood culture obtained at least 2 hours earlier in the catheter culture than the peripheral culture.

VIII 1.3. If 2 quantitative blood cultures are drawn from 2 catheter lumens; the colony count for the blood sample drawn through one lumen should beat least 3-fold greater than the colony count for the sample obtained from the second lumen.

VIII 1.4. If catheter removed: Same organism should be isolated from the peripheral blood culture and catheter tip culture. Catheter tip culture should have a positive result either by semi quantitative (growth of >15 colony forming units (cfu) from a 5-cm segment of the catheter tip) or by quantitative (growth of > 10^2^cfu by sonication method) method.

VIII 2. Recommendations for catheter management:

Once CRBSI is confirmed, the catheter should be removed if it was left in place and it should be reinserted at a new site, unless the organism is coagulase-negative *staphylococcus* or sensitive enterococcus and patient has shown improvement after starting antibiotics. In case of long-term catheters which are inserted in view of limited access; treatment can be attempted without catheter removal, with use of both systemic and antimicrobial lock therapy in case of uncomplicated CRBSI. If antibiotic lock therapy cannot be used, systemic antibiotics should be administered through the suspected lumen of the catheter. The catheter should be removed if blood cultures remain positive in spite of 72 hours of antimicrobial therapy.

VIII 3.0 -Recommendations for treatment of CRBSI

VIII 3.1. Empirical antibiotic therapy should be started promptly (preferably after obtaining blood cultures) when CRBSI is suspected. The local antibiogram should be considered while treating CRBSI. In general, coverage for both common gram-positive and gram-negative organisms is necessary [17].

VIII 3.2. Since staphylococci and enterococci are commonest organisms responsible for CRBSI; Vancomycin/Teicoplanin should be started as empirical therapy.

VIII 3.5. For carbapenem-resistant gram-negative organisms, treatment options include ceftazidime-avibactam plus aztreonam or polymyxins/colistin in combination with another agent demonstrating susceptible MIC [18, 19].

VIII 3.6. Empiric antifungal coverage should be given for patients with following risk factors: presence of femoral catheters, patients on total parenteral nutrition, prolonged use of broad-spectrum antibiotics or colonisation due to Candida species at multiple sites [20]. Echinocandins are favoured over azoles for empiric therapy as some candida species are resistant to azoles.

VIII 3.9. Usually, 10–14 days of antimicrobial therapy are required for treating uncomplicated CRBSI. For uncomplicated candidemia, antibiotic therapy should continue for 14 days after obtaining a negative blood culture.

VIII 3.10. 4–6 weeks of antibiotic/antifungal therapy should be administered to patients in whom fungaemia or bacteraemia persists even after 72 hours of catheter removal and to patients who are found to have infective endocarditis [21, 22, 17].

VIII 3.11. Teicoplanin has comparable efficacy to vancomycin, with the added advantage of lower nephrotoxicity [23]. Therefore, it can be a suitable alternative to vancomycin for first-line treatment for gram-positive infections. At our centre, teicoplanin is started as the first-line agent for suspected CRBSI.

## Question No- IX. What are the choices and duration of antifungals in invasive pulmonary aspergillosis (IPA)?

Answer:- Guidelines recommend considering empiric antifungal therapy in neutropenic fever when it is prolonged beyond 4–5 days [26]. However, efforts should be made to diagnose invasive fungal infections definitely when patients are on azole prophylaxis. There is a recent trend of increasing non-aspergillousmolds in severely immunocompromised patients which can change the choice and duration of antifungals. The initial choice for IPA is voriconazole which is based on RCT comparing voriconazole against amphotericin B deoxycholate demonstrated improved 12-week survival in the voriconazole treatment arm (71% versus 58%) [27]. When patients are on azole prophylaxis, the choice is between echinocandin or amphotericin-B based on type of organism and sensitivity. In patients with severe established fungal *pneumonia* with profound neutropenia, combination of echinocandin and voriconazole may be considered despite limited retrospective literature.

The duration of antifungals in IPA is usually 6–12 weeks based on expert recommendation. However, the duration also depends on severity, resolution and ongoing immunosuppression or neutropenia. Serial imaging can be considered; however, transient radiological worsening can be seen during recovery of neutrophil counts.

## Question No- X. What is the role of surveillance stool culture in guiding antibiotic decision?

Answer:-

The use of stool surveillance cultures to guide antibiotic selection has been proposed and practiced since late 1980s; however, robust literature confirming its utility is lacking [28]. However, robust literature confirming its utility is lacking. Data propounding its use is mainly retrospective in nature and limited in sample size. The largest data from India involving 313 allogeneic transplants in 299 patients showed a 56% incidence of MDR isolates in stool between 2014–2015 [29]. MDR isolation in stool was associated with higher incidence of 100 day mortality and higher incidence of MDR positivity in blood culture. However, a prospective study of 79 pediatric patients from PGIMER, India did not show an impact of MDR stool isolation on mortality or MDRO sepsis. Selection of antibiotics based on stool surveillance did not show any improvement in outcomes in this study [30]. Large retrospective data of 190 allogeneic transplant patients has shown that selecting first line antbitotics on basis of stool culture led to earlier defervescence of fever; however, there was no difference in infection-related mortality [31]. Similarly, a retrospective study of 317 transplant patients for benign diseases from 3 large pediatric bone marrow transplant centres from India did not any correlation between colonisation in rectal swab and clinical outcome. Antibiotic susceptibility testing didn’t correlate with *in vivo* clinical response [32].

Currently, there is limited evidence to recommend the use of stool surveillance culture to select first-line antibiotics. Hence, routine use of surveillance cultures or rectal swab for selection of antibiotics in immunocompromised patients is not recommended.

## Conflicts of interest

No conflicts of interests.

## Funding

No finances involved.

## Authors' contributions

Concept-MS, AK, VN, LN

Selection of questions- LN, GS, TG

Answers to the questions-LN, PS, AK, SS, BS, SB, AM, KB

Proofreading- SS, LN, BS, CD.

## Figures and Tables

**Table 1.** Neutropenia specific syndromes.

Syndrome	Diagnosis	Management
Neutropenic enterocolitis	1. Abdominal pain 2. USG abdomen showing bowel wall thickening >4 mm for more than 30 mm in length	1. Bowel rest 2. Antibiotics targetting enteric gram-negative bacteria, enterococci, and anaerobes. 3. Surgical evaluation
Invasive pulmonary Mold	1. Dry cough with pleuritic chest pain. 2. CT chest showing characteristic fungal nodules with GGO 3. Serum galactomanan	1. Choice of antifungals depends on type of antifungal used as prophyalxis
Invasive candidiasis	1. Cutaneous nodules 2. Hepatosplenic lesions 3. Raised serum Alkaline phosphatase	1. Echinocandins are preferred 2. Catheter related blood stream candidemia may require removal of central line.
Ecthyma Gangrenosum	1. Necrotic cutaneous lesion 2. Skin biopsy with culture of lesion 3. Common site- peripeneum and lower extremity	1. Surgical debridement 2. Broad spectrum antibiotic –gram positive and gram negative until culture report is ready.

**Table 2.** Empirical antibiotic therapy based on site of infection [24, 25].

Site	CAIs	HAIs
Bloodstream infection	IV: Ceftriaxone/Cefoperazone-Sulbactam	Piperacillin Tazobactam/Cefoperazone-Sulbactam/Imipenem/Meropenem/Ceftazidime-Avibactam (CZA) ± Vancomycin/Teicoplanin (if MRSA incidence is high).
Urinary tract infections (UTI)	Oral: Norfloxacin/Cefuroxime/Cefixime/AmoxicillinClavulanate/Nitrofurantoin/TrimethoprimSulfamethoxazole/Fosfomycin	IV: Ceftriaxone/Ofloxacin/Amikacin Imipenem/Meropenem/Piperacillin-Tazobactam/Cefoperazone-Sulbactam + Amikacin
Respiratory tract infections (RTI)	Oral: Amoxicillin-Clavulanate or Cefpodoxime ± Macrolide (Azithromycin) or Respiratory Fluoroquinolone(levofloxacin)	IV: Ceftriaxone or Amoxicillin-Clavulanate ± Macrolide. Imipenem/Meropenem/Piperacillin-Tazobactam/Cefoperazone-Sulbactam + Linezolid/Teicoplanin/Vancomycin ± Amikacin/Levofloxacin/Doxycycline/Minocycline.
Skin & soft tissue infections	Amoxicillin-Clavulanate or Cefuroxime/Cefadroxil ± Metronidazole/Clindamycin	Imipenem/Meropenem/Tigecycline/Piperacillin-Tazobactam/Cefoperazone-Sulbactam + Linezolid/Teicoplanin/Vancomycin ± Amikacin/Levofloxacin.
Intra-abdominal Infections	Oral: Ciprofloxacin with Ornidazole	IV: Ceftriaxone with Metronidazole Piperacillin Tazobactam/Cefoperazone-Sulbactam + Metronidazole

**Table 3.** Various methods for susceptibility testing from older to newer methods.

Methodology	Principle	Advantages	Limitations
Disk diffusion	Zone of inhibition measured around antibiotic disks.	Cost-effective, simple, standardized.	Lacks MIC determination, unreliable for certain resistance mechanisms (e.g., ESBLs, carbapenemases).
Broth micro dilution	MIC determined by observing bacterial growth in broth.	Quantitative, gold standard for MIC.	Time-intensive, requires manual preparation and technical expertise.
Agar dilution	MIC assessed by spotting bacteria on agar with antibiotic concentrations.	Accurate and reproducible for multiple strains.	Labor-intensive, not practical for routine diagnostics.
E test*	Gradient strip provides MIC based on inhibition ellipse.	Easy to use, provides quantitative MIC.	Expensive, results vary with agar and environmental factors.
Automated systems	Optical systems detect bacterial growth in micro dilution trays.	Rapid, high throughput, standardized.	High cost, limited resistance mechanism detection, potential for false susceptibility results.
Molecular methods	Detects resistance genes using PCR or sequencing.	Highly sensitive and specific, rapid.	Limited to known genes, lacks phenotypic correlation, and expensive.
MALDI-TOF MS**	Detects proteins or enzymatic activity linked to resistance.	Rapid results, low per-sample cost.	High initial investment, limited scope for novel resistance mechanisms.
Syndromic panels	Multiplex PCR identifies pathogens and resistance genes.	Comprehensive, rapid diagnosis.	Expensive, constrained to included targets, and no MIC determination.
NGS (Sequencing)	Comprehensive resistance profiling via whole-genome analysis.	Detects rare/novel mechanisms, high accuracy.	Costly, requires bioinformatics, time-intensive for routine use.

* E test-Epsilometer test

** MALDI-TOF -Matrix-Assisted Laser Desorption Ionisation–Time of Flight

**Table 4.** Indications of point-of-care testing for infections.

Indication	Description	Examples
Severe infections	Time-sensitive therapy required in critically ill patients.	Sepsis, ventilator-associated *pneumonia* (VAP), intra-abdominal infections.
High-risk populations	Patients at increased risk of resistant infections.	Cancer patients, transplant recipients, neutropenic individuals.
Empirical therapy failure	Cases where initial antibiotics are ineffective.	Patients unresponsive to first-line therapy due to undetected resistance genes.
Resource-limited settings	Rapid diagnostics in areas with limited access to advanced laboratory facilities.	Hospitals in low-resource settings with a high prevalence of antimicrobial resistance (AMR).
Post-surgical/Trauma care	Identifying resistance in infections associated with surgeries or trauma in healthcare settings.	Surgical site infections, hospital-acquired infections post-trauma.
Antimicrobial stewardship	Supporting targeted therapy and minimizing the use of broad-spectrum antibiotics.	Guiding appropriate antibiotic choices in multidrug-resistant infections.

**Table 5.** Overview of POC tests for resistance gene detection, highlighting their technology, features and clinical applications.

POC Test	Principle	Key features	Applications/ Indications
Cepheid GeneXpert	Cartridge based RT PCR	Detects KPC, NDM, VIM, OXA-48, and IMP Results in <1 hour User-friendly.	Detection of resistance genes from pure colonies.
BioFireFilmArray	Syndromic multiplex PCR	Comprehensive panels Detects IMP, KPC, OXA-48-like, NDM, VIM, CTX – M, mcr - 1 ~1-hour turnaround time	Identification of pathogens and resistance genes directly from patient’s sample (Blood, BAL, Sputum, Synovial fluid, CSF, Stool)
GenMark ePlex	Multiplex nucleic acid amplification	Similar to BiofireFilmArray ~1-hour turnaround time. Limited panels	Similar to BiofireFilmArray.
Abbott PLEX-ID	PCR-electrospray ionization mass spectrometry	Provides genotypic resistance profiles. Requires skilled personnel and laboratory infrastructure	Surveillance of resistance genes in high-risk and outbreak settings.
LAMP-based platforms	Loop-mediated isothermal amplification (LAMP)	Portable & cost-effective Lower specificity Limited resistance gene targets Risk of contamination	Detects carbapenemase genes but more used in resource-limited and field settings.
BD MAX system	Real-time PCR-based automation	~2-hour results. Limited portability Reagents are pricier	Detects carbapenemase genes but more useful in Infection control and outbreak management.
Verigene system	Microarray-based detection	~2.5-hour results. Limited Portability Reagents are pricier	Targeted AMR gene detection in bloodstream infections.
Resist 5 or Carba 5 test	Lateral flow immunoassay	Portable; rapid Detection (~30 minutes) Lower sensitivity & Specificity	Point-of-care and bedside. Diagnostics. Identifies resistance markers.
Isothermal PCR Platforms	Isothermal nucleic acid amplification	Simple workflow; detects AMR genes Results in <30 minutes Less accurate	Resource-limited and field settings.

**Table 6.** Resistance genes and preferred antibiotics.

Resistance gene	Resistance mechanism	Preferred antibiotics	Notes
Carbapenemases			
IMP	Metallo-β-lactamase (MBL), hydrolyzes carbapenems	Colistin, tigecycline, fosfomycin	Found in Gram-negative bacteria; often requires combination therapy.
KPC	*Klebsiella pneumoniae* carbapenemase	Ceftazidime-avibactam	Common in *Klebsiella* species; combination therapy may be needed.
OXA-48-like	Carbapenemase with weak activity against carbapenems	Ceftazidime-avibactam, fosfomycin	Prevalent in Enterobacterales; often found with additional resistance genes.
NDM	NDM	Colistin, tigecycline, Ceftazidime-avibactam plus aztreonam or cefiderocol	Highly resistant Gram-negatives; testing for combination therapies is crucial.
VIM	Verona integron-encoded metallo-β-lactamase	Colistin, fosfomycin, aztreonam	Common in *Pseudomonas* and other Gram-negatives; requires careful monitoring.
Colistin resistance			
mcr-1	Plasmid-mediated colistin resistance	Tigecycline, fosfomycin	A growing threat; colistin resistance significantly limits treatment options.
Extended spectrum beta lactamases			
ESBL (CTX-M, etc.)	Extended-spectrum β-lactamase	Carbapenems, TMP-SMX, Fluoroquinolones	CTX-M is a common ESBL; carbapenems remain the gold standard for severe infections.
Methicillin resistance			
mecA/C	Encodes PBP2a, leading to methicillin resistance	Vancomycin, linezolid, daptomycin	Found in MRSA and other *Staphylococcus* species; vancomycin is standard for severe cases.
Vancomycin resistance			
vanA/B	Glycopeptide resistance in Enterococcus species	Linezolid, daptomycin	vanA confers high-level resistance; vanB may show variable levels of resistance.
Others			
ermB	Methylation of ribosomal RNA, leading to macrolide (Erythromycin, azithromycin) resistance	Clindamycin (if D-test negative), beta lactams, fluoroquinolones	Found in *Streptococcus* species; careful testing needed to confirm clindamycin efficacy.
tetK	Efflux pump conferring tetracycline resistance	Doxycycline, minocycline, drugs from other classes	Found in *Staphylococcus* species; doxycycline often remains effective.
gyrA mutations	Confers fluoroquinolone resistance	Amikacin, beta-lactams, Fosfomycin	Often seen in *Salmonella* and *E. coli*; alternatives depend on susceptibility profiles.

**Table 7.** Various methods for *C difficile* detection.

Testing modality	Sensitivity	Specificity	Advantages	Limitations
Cell culture cytotoxicity neutralization assay (CCCNA)	65%–90%	High	Detects toxin-induced cytopathic effects; considered historical gold standard	Time-consuming (24–48 hours), variable sensitivity, requires expertise in cell culture
Toxigenic culture	High	High	Highly sensitive; crucial for epidemiological studies	Labor-intensive, slow turnaround (up to 7 days), impractical for routine diagnostics
Enzyme immunoassays (EIAs)	40%–80%	Moderate to High	Rapid and cost-effective	Low sensitivity, frequent false negatives; unsuitable as standalone test
Glutamate dehydrogenase (GDH)	80%–100%	Low	High sensitivity; rapid screening for *C. difficile* presence	Cannot distinguish toxigenic from non-toxigenic strains; requires confirmatory testing
Nucleic acid amplification tests (NAATs)	High	High	Rapid, highly sensitive and specific; standalone diagnostic tool	Detects colonization, potentially leading to overtreatment
Combination Testing (e.g., GDH + Toxin EIAs/NAATs)	High	High	Balances cost, accuracy, and speed; efficient diagnostic algorithm	Requires sequential testing and integration of multiple methodologies

In conclusion, NAATs are the most reliable standalone test, offering high sensitivity and specificity. Combination testing using GDH screening followed by toxin assays or NAATs is cost-effective and ensures diagnostic accuracy, particularly in resource-limited settings.

## Appendix-A

**Clinical scenario 1:** Indications for point-of-care resistant gene testing in GNB.

Parameter	Details
Patient	62/ M
Underlying condition	Relapsed case of Acute Myeloid Leukemia (AML) on day 10 of induction chemotherapy
Presentation	High-grade fever (39.4°C), hypotension (BP 84/50 mmHg), tachycardia, abdominal pain and in neutropenic shock
Neutrophil count	ANC: 40 cells/μl
Past medical history	Previous ICU admissions; colonized with MDR *K. pneumoniae*
Prior antibiotic exposure	Multiple courses of Carbapenems and Colistin
Local epidemiology	High prevalence of NDM/OXA-48 in ICU isolates
Investigations ordered	Blood cultures (2 sets) Urine, stool, central line cultures CBC, CRP, PCT, LFTs, RFTs Serum lactate: 3.4 mmol/l CECT abdomen
Molecular test	**Point-of-care resistant gene panel i.e. *Xpert Carba-R* from the growth**
Gene detected	**NDM-positive**
Diagnosis	Neutropenic septic shock likely due to NDM-producing Gram-negative bacteremia
Clinical decision	Empiric therapy escalated to **Aztreonam + Ceftazidime - Avibactam** based on gene detection
Rationale for testing	Rapid clinical deterioration Prior CRE colonization High local prevalence of resistance genes Delayed culture results Need for early effective therapy

**Clinical scenario 2:** Susceptibility testing in gram-negative infections.

Parameter	Details
Patient	45/ F
Clinical background	Carcinoma cervix on chemotherapy, admitted with fever and dysuria. Past history of UTI which was treated with ceftriaxone 3 weeks ago, empirically from a local practitioner.
Presentation	Fever (38.8°C), flank pain, pyuria
Preliminary diagnosis	Febrile neutropenia with suspected **urosepsis**
Initial labs	ANC: 750/mm³, PCT: 2.1 ng/ml
Empiric antibiotics	Started on **Piperacillin-Tazobactam.**
Culture result	***Enterobacter cloacae*** isolated from urine and blood.
Susceptibility method used	VITEK-2 (automated system)
Initial report	Sensitive to ceftriaxone, piperacillin-tazobactam, cefepime, meropenem
Problem faced	Patient deteriorated despite on piperacillin-tazobactam.
Action taken	The final identification- *Enterobacter cloacae* is an inducible Amp-C producer. Patient can be escalated to either a fourth generation cephalosporin (carbapenem sparing option) or a carbapenem.
Revised treatment	Switched to **Cefepime** based on availability and susceptibility and this was a relatively stable patient. Cefepime is more stable against AmpC enzymes
Clinical outcome	Gradual improvement over 7 days; afebrile and subsequent follow-up blood cultures were negative and patient was discharged.
Learning points	Enterobacter cloacae is an inducible Amp-c producer. Organisms like *Citrobacter freundii*, *Enterobacter cloacae*, and *Serratia marcescens, Proteus vulgaris* (SPiCE group of organisms) produce **inducible AmpC enzymes,** which are **NOT reliably inhibited by β-lactamase inhibitors** and may be **induced during cephalosporin therapy**, leading to **treatment failure** despite initial susceptibility.
